# Autonomous Navigation of a Team of Unmanned Surface Vehicles for Intercepting Intruders on a Region Boundary

**DOI:** 10.3390/s21010297

**Published:** 2021-01-04

**Authors:** Ali Marzoughi, Andrey V. Savkin

**Affiliations:** 1 School of Electrical Engineering and Industrial Automation, Engineering Institute of Technology, Wellington St, West Perth, WA 6005, Australia; a.marzoughi@eit.edu.au; 2 School of Electrical Engineering and Telecommunications, The University of New South Wales, Sydney, NSW 2052, Australia

**Keywords:** unmanned surface vehicles, navigation, USVs, autonomous systems, robot control, mobile robots, autonomous vehicles, multi-agent systems, border protection, interception, distributed robotic networks, guidance, multi-robot systems, decentralised navigation algorithms, decentralised sliding mode control

## Abstract

We study problems of intercepting single and multiple invasive intruders on a boundary of a planar region by employing a team of autonomous unmanned surface vehicles. First, the problem of intercepting a single intruder has been studied and then the proposed strategy has been applied to intercepting multiple intruders on the region boundary. Based on the proposed decentralised motion control algorithm and decision making strategy, each autonomous vehicle intercepts any intruder, which tends to leave the region by detecting the most vulnerable point of the boundary. An efficient and simple mathematical rules based control algorithm for navigating the autonomous vehicles on the boundary of the see region is developed. The proposed algorithm is computationally simple and easily implementable in real life intruder interception applications. In this paper, we obtain necessary and sufficient conditions for the existence of a real-time solution to the considered problem of intruder interception. The effectiveness of the proposed method is confirmed by computer simulations with both single and multiple intruders.

## 1. Introduction

The variety of real-time applications of multi-robot teams has been dramatically increasing due to the rapid development in robotics technology. Therefore, the use of autonomous mobile robots has been increased significantly in real-time applications such as border surveillance, patrolling and monitoring. Navigation and control of a multi-robot team for patrolling the border of a region is a fundamental problem of multi-agent robotics [1,2,3,4]. The coverage control approach is known as the most prevalent method to protect an environment from unwanted intruders where both the problems of sweep and barrier coverage control are studied. In the barrier coverage strategy, a mobile robotic network with sensing capability is deployed to form a barrier of static sensors. Each robot of the team detects any unwanted intrusion that occurs by an intruder on the boundary of the region [5,6]. On the other hand, the sweeping coverage is a strategy for a multi robot team which sweeps along the border of a protected region. Therefore, in any time *t*, some robots detect every point in some neighbourhood of the boundary [7,8].

In this study, we consider a planar region where each agent of a multi-robot team has to move along the border of the region to protect the most vulnerable point of the boundary. The navigation strategy is decentralised, as the only available information for each robot is the coordinates of its current position and the coordinates of the current positions of a few of the closest team-mates, which are collected by onboard sensors; therefore, each robot navigates independently along the boundary. Such a multi-robot team is quite a typical example of a multi-agent system; see, for example [5,6,7,8,9,10,11]. Intercepting an intruder or several intruders is the objective of the team of mobile robots, in a way that at least one robot must be close to the intrusion point when the intruder is about to cross the boundary of the protected region. Moreover, in this paper we concentrate on navigation of a team of autonomous unmanned surface vehicles (USVs); see, for example [12,13]. Real life examples of the investigated problem are various asset guarding problems. In such problems, a team of autonomous USVs is responsible for guarding the asset that is threatened by hostile boats, by cooperatively patrolling the surrounding region of the asset to actively detect, identify and block any invasive intruders [14,15,16]. Guarding of the civilian harbours from unexpected terrorist attacks, which can possibly occur in the “blue border” (i.e., the sea-side), is an important example of this problem [17]. Another real life application of the problem of autonomous navigation of a team of USVs for intercepting intruders on a region boundary is protecting swimmers and surfers from shark attacks [18].

In this paper, we propose a decentralised navigation control strategy for a group of autonomous USVs with a necessary and sufficient condition, which guaranties intercepting the intruder in the boundary of the region. The decentralised navigation control strategy proposed in this paper does not require complex computations and it is easily implementable in real-time.

The remainder of the paper is organised as follows. Section 2 gives a brief survey of the existing literature in the field that is related to the topic of the current paper. The main definitions and the problem statement are formulated in Section 3. Section 4 presents the proposed autonomous navigation algorithm and its mathematical analysis. In Section 5, the validation of the proposed navigation strategy is conducted by computer simulations and illustrative examples. Finally, a brief conclusion is presented in Section 6.

## 2. Related Work

In this section, CP is short for Coverage Problem.Three types of coverage problems for mobile robotic sensor networks are defined in the well-known survey article by Gage [19], namely:

CP1. Barrier coverage: to achieve a static arrangement of robotic sensors that minimises the probability of undetected intrusion through the barrier; see, for example [5,6,8].

CP2. Sweep coverage: to move a number of mobile robots across a sensing field so that it addresses a specified balance between maximising the detection rate of events and minimising the number of missed detections per unit area; see, for example [6,7,8,9].

CP3. Blanket coverage: to achieve a static arrangement of robotic sensors that maximises the detection rate of targets appearing in the sensing field; see, for example [6,20].

In barrier coverage, a sensing barrier is formed by an array of mobile robots equipped with sensors so that any intrusion through the barrier is detected. Sweep coverage is achieved by moving a number of mobile robots across a sensed field to search for and detect targets in the field. Finally, the purpose of blanket coverage is to monitor a given area so that targets appearing in this area are detected by the network of mobile robots equipped with sensors.

The approach suggested in this paper can be viewed as a dynamic version of the barrier coverage problem. Unlike the static barrier coverage approach of [5,6,8] where the mobile sensors move along the boundary of a region to reach some static positions without taking into account the intruders’ motion, in the proposed approach, the autonomous robots move along the boundary always reacting to intruders’ motion. Notice that a dynamic version of the blanket coverage problem with unmanned aerial vehicles was considered in [21].

The problem with a single intruder and a single intercepting autonomous vehicle was referred to in [22] as the “Lady in the lake problem". The reactive navigation algorithm proposed in [22] is based on a pursuit-evasion game including a single intruder and a single interceptor. The case of an individual intruder and a couple of interceptors that are protecting the environment by moving on the boundary of a rectangular region was studied in [23]. Furthermore, a scenario with *n* intruders and *n* defenders was studied in [24]. A problem with several defenders and an intruder team trying to score by sending as many intruders as possible to the target was studied in [25]. In the problem of [25], the defenders cannot enter the target region but they can move outside its perimeter.

The publication [26] considered a problem of navigating a network of aerial drones along the frontier of an environmental disaster area so that they are able to monitor the faster moving segment of the frontier. A feature of the approach of [26] is that the region is moving and deforming unlike in the problems studied in [5,6,7,8]. The main application of the method of [26] is an important problem of bushfire monitoring (see, e.g., [27]) as well as the problem of oil spill monitoring and mapping [28].

In this paper, we consider the problems with several defenders and a single intruder and with several defenders and several intruders, where the defenders can move only along the region boundary. Unlike coverage control problems of [5,6,8], where the decentralised control algorithms for mobile robotic sensor networks eventually steered the mobile sensors into some uniformly distributed stationary positions or trajectories, the algorithm developed in this paper results in a quite dynamic motion of a team of autonomous USVs where the positions of the autonomous USVs always depend on the intruder’s behaviour in some time $t_{0}$, when, the intruder becomes visible to the USVs, i.e., the planar coordinates of the intruder are known by all the team members after a certain time moment. Unlike the publications [5,6,7,8,9] where mobile ground robots were considered and [21,26] coverage problems with autonomous aerial drones were studied, this paper concentrates on a team of autonomous USVs. Navigating of autonomous USVs is an active area of research of which importance is quickly growing [29,30,31,32,33].

## 3. Problem Statement

**Figure 1:**
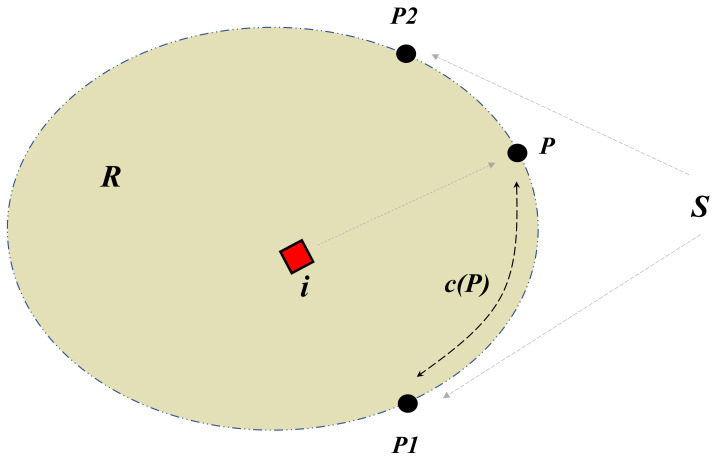
R: The closed convex planar region, S: The segment of the boundary needs to be protected, c(P): The curvilinear coordinate.

The region R is considered to be a planar closed convex region that is surrounded by a smooth and a piecewise boundary. Notice that R may be unbounded. Furthermore, as shown in Figure 1, S is a segment of the region R that is located between two given points $P_{1}$ and $P_{2}$. Based on the scenario, a moving point-wise intruder that is trapped inside the region R strives to escape the terrain through any point of the segment S; see Figure 1. We assume that the intruder cannot cross the boundary of R outside the segment S. We define $x_{I}\left(t\right)$ as the point-wise intruder’s planar coordinates. The intruder is supposed to move arbitrarily inside the region R with a vector velocity $v_{I}\left(t\right)={\dot{x}}_{I}\left(t\right)$ that is a time varying vector and satisfies the constraint:

(1) $$∥v_{I}\left(t\right)∥\leq V_{I}^{max}\;\;\;\;\forall t\geq 0$$

where $V_{I}^{max}>0$ is supposed to be constant and ∥·∥ represents the standard Euclidean vector norm. Also, let $x_{I}\left(t\right)$ be the planar coordinates of the intruder *I* at time *t*. Moreover, let *n* be an integer that satisfies n>1 and denotes the number of autonomous USVs in the multi-USV team.

Then, the point-wise autonomous USVs are labelled as 1,2,…,n, and they prevent the intruder from leaving the region R through the segment S. In this scenario, the USVs are only able to move along the segment S; however, the invasive intruder is supposed to be a multi directional moving object in the terrain. The planar coordinates of each USV 1,2,…,n are denoted as $x_{1}\left(t\right),x_{2}\left(t\right),\ldots ,x_{n}\left(t\right)$.

Furthermore, c(P) is defined as the curvilinear coordinates of any point P∈S, which represents the length of any sub-segment $(P,P_{1})\in S$.; see Figure 1. If the length of the segment S is supposed to be *L*, then this implies that $c(P_{1})=0$ and $c(P_{2})=L$. We assume that at time t≥0, the curvilinear coordinate of each USV (1,2,…,n) is represented by

$$(c_{1}\left(t\right):=c\left(x_{1}\left(t\right)\right),c_{2}\left(t\right):=c\left(x_{2}\left(t\right)\right),\ldots ,c_{n}\left(t\right):=c\left(x_{n}\left(t\right)\right).$$

Furthermore, the team members are labelled based on their curvilinear coordinates such that:

(2) $$\begin{array}{c} 0\leq c_{1}\left(t\right)\leq c_{2}\left(t\right)\leq \ldots \leq c_{n}\left(t\right)\leq L\;\;\;\forall t\geq 0. \end{array}$$

Based on the requirement (2), none of the team members are allowed to change the formation order of the team on S⊂R. Equation (3) describes the way the USVs move along segment S on the boundary.

(3) $$\begin{array}{c} {\dot{c}}_{i}\left(t\right)=u_{i}\left(t\right)\;\;\;\forall k=1,2,\ldots ,n \end{array}$$

In Equation (3), $u_{i}\left(t\right)$ represents the control input of the USV *i*, which should satisfy the constraint

(4) $$|u_{i}\left(t\right)|\leq V_{R}^{max}\;\;\;\;\forall t\geq 0$$

where $V_{R}^{max}>0$ is a given constant.

Available measurements: Any USV i,2≤i≤n−1 knows the curvilinear coordinates of its own $c_{i}\left(t\right)$ as well as the curvilinear coordinates $c_{i-1}\left(t\right)$, $c_{i+1}\left(t\right)$ of the USVs i−1 and i+1, at any time *t*. The USVs 1 and *n* know the curvilinear coordinates $c_{2}\left(t\right)$, $c_{n-1}\left(t\right)$ of the USVs 2 and n−1, respectively. The team members detect the intruder at a time $t_{0}\geq 0$, which means that for $t\geq t_{0}$, all team members know the planar coordinates $x_{I}\left(t\right)$ of the intruder.

Notice that this assumption usually holds in asset protection problems where a high risk region containing highly significant assets has been guarded by a team of autonomous unmanned surface marine vehicles (USVs) and where a hostile boat becomes visible to the marine vehicles of the team approximately at the same time [14,15,16,17]. Such situations are also in problems where defence robots and an intruder are aerial drones; see, for example [34]. The paper does not discuss how the intruders are detected, as it concentrates on navigation issues and detection issues are beyond its scope. In practice, detection of intruders can be done using radar technology or video cameras.

**Definition** **1.** *We consider a given positive constant ϵ>0. Furthermore, we suppose that the trapped intruder aims to escape the region R at time $t_{⋆}$ by crossing the segment S, i.e., $x_{I}\left(t_{⋆}\right)\in S$. Therefore, if there exists any USV i (for i=1,2,…,n), in which $|c\left(x_{I}\left(t_{⋆}\right)\right)-c_{i}\left(t_{⋆}\right)|\leq \epsilon$, the intruder has been ϵ-intercepted by the team of autonomous USVs. The proposed navigation strategy is called ϵ-intercepting strategy, if at least one of the team members ϵ-intercepts the intruder while the intruder is about to cross the segment S through the interception point.*

Escaping the region R by crossing the boundary through any interception point P∈S⊂R without being ϵ-intercepted by any individual member of the team of interceptors is the main objective of the intruder.

The main objective in this paper is to derive a necessary and sufficient condition for existence of a decentralised navigation control strategy, based on available information for the team of autonomous USVs which achieves ϵ-intercepting the intruder or several intruders for any intruders’ motion. Furthermore, another objective is to develop a computationally efficient and easily implementable in real-time ϵ-intercepting strategy for the USV team.

Notice that this problem can be viewed as a problem of decentralised control of a multi-agent system; see, for example [35,36].

## 4. Main Techniques

### 4.1. Navigation Algorithm

Suppose that P∈S and *x* is a point inside the region R. Then L(x,P) represents a straight line segment connecting *x* and *P*. Since R is convex, L(x,P) is in R and *P* is the only intersection point of L(x,P) and the boundary of R. Moreover, α(x,P) is supposed to be the length of L(x,P). Furthermore, let *i* be a an index such that

$$|c_{i}\left(t\right)-c\left(P\right)|\leq |c_{j}\left(t\right)-c\left(P\right)|\;\;\;\;\forall j=1,\ldots ,n.$$

Then, we define the length of a sub-segment of the segment S, which denotes the distance between the current location of the closest USV *i* to the interception point *P* at time *t* by a variable $\beta \left(t,P\right):=|c_{i}\left(t\right)-c\left(P\right)|$.

We define F(s) as a function from the interval [0,L] where s∈[0,L] is a number. Then we can say that, for any s∈[0,L], F(s)=P∈S, such that c(P)=s.

Furthermore, we consider a subsegment $[A_{1},A_{2}]\in S$, which is closed by points $A_{1}$ and $A_{2}$. For i=1,…,n, we introduce sub-segments $[S_{i}{\left(t\right)}^{-},S_{i}{\left(t\right)}^{+}]\in S$ as follows:

(5) $$\begin{array}{c} S_{i}{\left(t\right)}^{-}:=\left[F\left(\frac{c_{i-1}\left(t\right)+c_{i}\left(t\right)}{2}\right),F\left(c_{i}\left(t\right)\right)\right]\\ if\;\;\;i=2,\ldots ,n;\\ S_{i}{\left(t\right)}^{-}:=\left[P_{1},F\left(c_{i}\left(t\right)\right)\right]\\ if\;\;\;i=1;\\ S_{i}{\left(t\right)}^{+}:=\left[F\left(c_{i}\left(t\right)\right),F\left(\frac{c_{i}\left(t\right)+c_{i+1}\left(t\right)}{2}\right)\right]\\ if\;\;\;i=1,\ldots ,n-1;\\ S_{i}{\left(t\right)}^{+}:=\left[F\left(c_{i}\left(t\right)\right),P_{2}\right]\\ if\;\;\;i=n. \end{array}$$

Moreover, for i=1,2,…,n, introduce the numbers $M_{i}^{-}\left(t\right)$ and $M_{i}^{+}\left(t\right)$ as

(6) $$\begin{array}{c} M_{i}^{-}\left(t\right):=\underset{P\in S_{i}{\left(t\right)}^{-}}{\sup}\left(\beta \left(t,P\right)-\frac{\alpha \left(x_{I}\left(t\right),P\right)V_{R}^{max}}{V_{I}^{max}}\right);\\ M_{i}^{+}\left(t\right):=\underset{P\in S_{i}{\left(t\right)}^{+}}{\sup}\left(\beta \left(t,P\right)-\frac{\alpha \left(x_{I}\left(t\right),P\right)V_{R}^{max}}{V_{I}^{max}}\right). \end{array}$$

Now the decentralised control navigation law for the autonomous USVs is proposed as follows:

(7) $$\begin{array}{c} u_{i}\left(t\right):=V_{R}^{max}\;\;\;if\;\;\;M_{i}^{-}\left(t\right)<M_{i}^{+}\left(t\right)\\ u_{i}\left(t\right):=-V_{R}^{max}\;\;\;if\;\;\;M_{i}^{-}\left(t\right)>M_{i}^{+}\left(t\right)\\ u_{i}\left(t\right):=0\;\;\;if\;\;\;M_{i}^{-}\left(t\right)=M_{i}^{+}\left(t\right) \end{array}$$

for all i=1,…,n.

**Remark** **1.** *The following explanation of the navigation law (7) gives a clearer perception behind it. At time t, $[S_{i}{\left(t\right)}^{-},S_{i}{\left(t\right)}^{+}]\in S$, represents a set of closest points of segment S to the USV i. Depending on which of them is more “dangerous" with its maximum allowed speed at time t, the team member i drives through either the segment $S_{i}{\left(t\right)}^{-}$ or the segment $S_{i}{\left(t\right)}^{+}$. The term “dangerous" is applied to either of them ($S_{i}{\left(t\right)}^{-}$ or $S_{i}{\left(t\right)}^{+}$) with the maximum possible distance from the intruder to the USV i while the intruder is about to cross the segment S at interception point. The maximum possible distance between the closest interceptor of the team and the invasive intruder is described by (6).*

**Remark** **2.** *The navigation law (7) belongs to the class of sliding mode control laws where switching between two or more continuous controllers occurs; see, for example*, [37,38,39].

### 4.2. Mathematical Analysis of the Navigation Algorithm

**Theorem** **1.** *Suppose that the conditions (1) and (4) are satisfied for the team of autonomous USVs (3) and the intruder I. Then a decentralised ϵ-intercepting navigation strategy exists for the team of autonomous USVs if and only if:*

(8) $$\underset{P\in S}{\sup}\left(\beta \left(t_{0},P\right)-\frac{\alpha \left(x_{I}\left(t_{0}\right),P\right)V_{R}^{max}}{V_{I}^{max}}\right)\leq \epsilon$$

*where $t_{0}\geq 0$ represents the moment at which the interceptors see the intruder. Furthermore, if the inequality (8) holds, then the navigation law (7) is an ϵ-intercepting navigation strategy.*

**Remark** **3.** *Notice that since the region R is convex, and the segment S is compact, the supremum in (8) is achieved for some point P.*

**Proof of Theorem** **1.** At the first stage, we consider the case that the inequality (8) does not hold, in this case we prove that the intruder can always cross the segment S without ϵ-intercepting by the team of autonomous USVs. Indeed, if (8) does not hold, then there exists a point P∈S such that

(9) $$\left(\beta \left(t_{0},P\right)-\frac{\alpha \left(x_{I}\left(t_{0}\right),P\right)V_{R}^{max}}{V_{I}^{max}}\right)>\epsilon .$$

Now let the intruder move along the straight line segment $L(x_{I}\left(t_{0}\right),P)$ connecting the points $x_{I}\left(t_{0}\right)$ and *P* with its maximum speed $V_{I}^{max}$. In this case, the intruder reaches the point *P* at the time

$$t_{*}=t_{0}+\frac{\left|L(x_{I}\left(t_{0}\right),P)\right|}{V_{I}^{max}}.$$

It obviously follows from (9), that the closest USV to the point *P* cannot be closer to *P* at the time $t_{*}$ than ϵ. Therefore, the ϵ-neighbourhood of the point $P=x_{I}\left(t_{*}\right)$ at the segment S cannot contain any USV at time $t_{*}$. This implies that the team of autonomous USVs does not ϵ-intercept the intruder.

Then we prove that if the inequality (8) holds, the team of autonomous USVs navigated by the law (7) always ϵ-intercepts the intruder when it crosses the segment S. First, we prove the following claim. Indeed, we introduce the Lyapunov function (see, e.g., [40]) for any trajectory $\left[x_{I}\left(t\right),c_{1}\left(t\right),\ldots ,c_{n}\left(t\right)\right]$ of the USV-interceptors-intruder system as follows:

(10) $$\begin{array}{c} W[x_{I}\left(t\right),c_{1}\left(t\right),\ldots ,c_{n}\left(t\right)]:=\\ \underset{P\in S}{\sup}\left(\beta \left(t,P\right)-\frac{\alpha \left(x_{I}\left(t\right),P\right)V_{R}^{max}}{V_{I}^{max}}\right). \end{array}$$

Notice that since the region R is convex, and the segment S is compact, the supremum in (10) is achieved for some point *P*. Furthermore, by definition, $\alpha (x_{I}\left(t\right),P)$ is the length of the straight segment $L(x_{I}\left(t\right),P)$ connecting $x_{I}\left(t\right)$ and *P*. Hence, it is obvious that

(11) $$\alpha \left(x_{I}\left(t\right),P\right)=\underset{M\left(x_{I}\left(t\right),P\right)\in M\left(x_{I}\left(t\right),P\right)}{\inf}\left|M\left(x_{I}\left(t\right),P\right)\right|$$

where $M(x_{I}\left(t\right),P)$ is the set of all smooth paths $M(x_{I}\left(t\right),P)$ inside R connecting $x_{I}\left(t\right)$ and *P*, and $\left|M(x_{I}\left(t\right),P)\right|$ denotes the length of the path $M(x_{I}\left(t\right),P)$. In other words, $M(x_{I}\left(t\right),P)$ is the set of all possible paths of the intruder between $x_{I}\left(t\right)$ and *P*. Furthermore, it immediately follows from (10), (11) and (7) that

(12) $$\begin{array}{c} W[x_{I}\left(t_{2}\right),c_{1}\left(t_{2}\right),\ldots ,c_{n}\left(t_{2}\right)]\leq \\ W\left[x_{I}\left(t_{1}\right),c_{1}\left(t_{1}\right),\ldots ,c_{n}\left(t_{1}\right)\right]\;\;\;\forall t_{2}>t_{1}\geq t_{0}. \end{array}$$

Now (12) and (9) imply that if the intruder reaches a point P∈S at some time $t_{*}\geq t_{0}$, the closest USV to the point *P* at time $t_{*}$ cannot be further from *P* than ϵ. Therefore, the ϵ-neighbourhood of the point $P=x_{I}\left(t_{*}\right)$ at the segment S contains at least one USV at time $t_{*}$. This implies that the team of the interceptors ϵ-intercepts the intruder and the Theorem 1 has been proved. □

Now, we simplified the inequality (8) and the navigation law (7) by considering Assumption 1 as follows:

**Assumption** **1.** *The following inequality holds:*

$$V_{I}^{max}\geq V_{R}^{max}.$$

Then for i=k,k+1,…,n−k+1. First we introduce some points $D_{i}{\left(t\right)}^{-},D_{i}{\left(t\right)}^{+}\in S$ as follows:

(13) $$\begin{array}{c} D_{i}{\left(t\right)}^{-}:=F\left(\frac{c_{i-1}\left(t\right)+c_{i}\left(t\right)}{2}\right)\;\;\;\\ if\;\;\;i=2,\ldots ,n;\\ D_{i}{\left(t\right)}^{-}:=P_{1}\;\;\;if\;\;\;i=1; \end{array}$$

(14) $$\begin{array}{c} D_{i}{\left(t\right)}^{+}:=F\left(\frac{c_{i}\left(t\right)+c_{i+1}\left(t\right)}{2}\right)\;\;\;\\ if\;\;\;i=1,\ldots ,n-1; \end{array}$$

(15) $$\begin{array}{c} D_{i}{\left(t\right)}^{+}:=P_{2}\;\;\;if\;\;\;i=n. \end{array}$$

Second, we introduce numbers $H_{i}^{-}\left(t\right)$ and $H_{i}^{+}\left(t\right)$ as follows:

(16) $$\begin{array}{c} H_{i}^{-}\left(t\right):=\frac{c_{i}\left(t\right)-c_{i-1}\left(t\right)}{2}-\frac{\alpha \left(x_{I}\left(t\right),D_{i}{\left(t\right)}^{-}\right)V_{R}^{max}}{V_{I}^{max}}\\ if\;\;\;i=2,\ldots ,n;\\ H_{i}^{-}\left(t\right):=c_{i}\left(t\right)-\frac{\alpha \left(x_{I}\left(t\right),P_{1}\right)V_{R}^{max}}{V_{I}^{max}}\\ if\;\;\;i=1;\\ H_{i}^{+}\left(t\right):=\frac{c_{i+1}\left(t\right)-c_{i}\left(t\right)}{2}-\frac{\alpha \left(x_{I}\left(t\right),D_{i}{\left(t\right)}^{+}\right)V_{R}^{max}}{V_{I}^{max}}\\ if\;\;\;i=1,\ldots ,n-1;\\ H_{i}^{+}\left(t\right):=L-c_{i}\left(t\right)-\frac{\alpha \left(x_{I}\left(t\right),P_{2}\right)V_{R}^{max}}{V_{I}^{max}}\\ if\;\;\;i=n. \end{array}$$

Then, the navigation law (7) becomes simplified (for i=1,…,n) as follows:

(17) $$\begin{array}{c} u_{i}\left(t\right):=V_{R}^{max}\;\;\;if\;\;\;H_{i}^{-}\left(t\right)<H_{i}^{+}\left(t\right)\\ u_{i}\left(t\right):=-V_{R}^{max}\;\;\;if\;\;\;H_{i}^{-}\left(t\right)>H_{i}^{+}\left(t\right)\\ u_{i}\left(t\right):=0\;\;\;if\;\;\;H_{i}^{-}\left(t\right)=H_{i}^{+}\left(t\right). \end{array}$$

**Theorem** **2.** *Suppose that the conditions (1), (4) and Assumption 1 hold for the team of autonomous USVs (3) and the intruder I. Furthermore, H is supposed to be a set of numbers $H_{i}^{-}\left(t_{0}\right),H_{i}^{+}\left(t_{0}\right)$ in which i=k,k+1,…,n−k+1 and the intruder I, becomes visible to the team members (interceptors) at some time $t_{0}\geq 0$. Then there exists an ϵ-intercepting navigation strategy for the team of interceptors if and only if*

(18) maxH≤ϵ.

*Furthermore, if the inequality (18) holds, the navigation law (17) is an ϵ-intercepting navigation strategy.*

**Proof of Theorem** **2.** We prove that if Assumption 1 holds, then

(19) $$\begin{array}{c} M_{i}^{-}\left(t\right)=H_{i}^{-}\left(t\right),\;\;\;M_{i}^{+}\left(t\right)=H_{i}^{+}\left(t\right) \end{array}$$

where $M_{i}^{-}\left(t\right),M_{i}^{+}\left(t\right),H_{i}^{-}\left(t\right),H_{i}^{+}\left(t\right)$ are defined by (6), (16). Indeed, let $P_{3},P_{4}\in S_{i}{\left(t\right)}^{-}$ and $c\left(P_{3}\right)<c\left(P_{4}\right)$ where $S_{i}{\left(t\right)}^{-}$ is defined by (5). Then, for any *x*, we obviously have that

$$\alpha \left(x,P_{3}\right)\leq \alpha \left(x,P_{4}\right)+c\left(P_{4}\right)-c\left(P_{3}\right).$$

This and Assumption 1 imply that

$$\begin{array}{c} \beta \left(t,P_{3}\right)-\frac{\alpha \left(x,P_{3}\right)V_{R}^{max}}{V_{I}^{max}}\geq \beta \left(t,P_{4}\right)-\frac{\alpha \left(x,P_{4}\right)V_{R}^{max}}{V_{I}^{max}} \end{array}$$

for any *x*. This implies that

$$\underset{P\in S_{i}{\left(t\right)}^{-}}{\sup}\left(\beta \left(t,P\right)-\frac{\alpha \left(x,P\right)V_{R}^{max}}{V_{I}^{max}}\right)$$

is achieved at the left end of the interval $S_{i}{\left(t\right)}^{-}$. Therefore, $M_{i}^{-}\left(t\right)=H_{i}^{-}\left(t\right)$. Analogously, $M_{i}^{+}\left(t\right)=H_{i}^{+}\left(t\right)$. Hence, (19) holds and the statement of Theorem 2 follows from Theorem 1. Therefore, Theorem 2 has been proved. □

### 4.3. Comparison with the Static Barrier Coverage Approach

**Figure 2:**
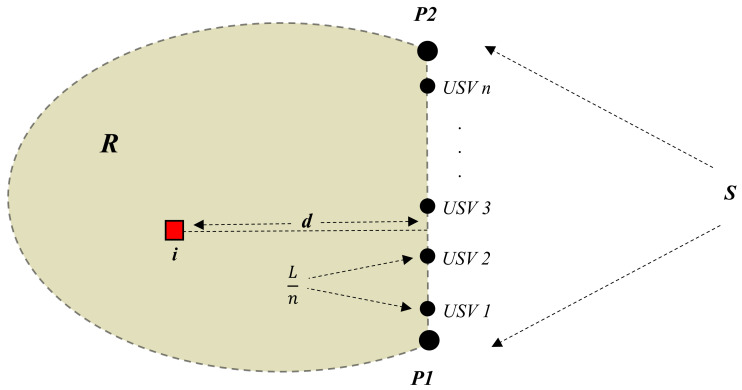
Deployment of autonomous unmanned surface vehicles (USVs) on a straight line segment S.

To make a comparison of the proposed method to the static barrier coverage approach of [5], we consider the following illustrative example. Let the segment S be a straight line segment, and assume that the *n* USVs are equally spaced on this segment, so that the distances between two neighbouring USVs are $\frac{L}{n}$ and the distances between the USV 1 and the USV n and the ends of the segment S are $\frac{L}{2n}$. Furthermore, assume that at the time $t_{0}$ the intruder is located at the line orthogonal to the middle of the segment interval between two neighbouring USVs at the distance *d* from this middle point, see Figure 2.

It is obvious that if the USVs are static as in [5], then the USVs are able to ϵ-intercept the intruder if and only if $\frac{L}{2n}\leq \epsilon$. On the other hand, it obviously follows from Theorem 2, that if Assumption 1 holds, then there exists an ϵ-intercepting navigation strategy for the team of USVs if and only if

(20) $$\begin{array}{c} \frac{L}{2n}\leq \epsilon +\frac{dV_{R}^{max}}{V_{I}^{max}}. \end{array}$$

Furthermore, if the inequality (20) holds, the navigation law (17) is an ϵ-intercepting navigation strategy. Notice that (20) can be viewed as some objective metrics describing the performance of the proposed method as a function of $L,n,d,V_{R}^{max},V_{I}^{max}$. This example shows that the proposed dynamic barrier coverage method has a significant advantage over the static barrier coverage approach of [5].

### 4.4. The Case of Several Intruders

The problem of a single intruder interception by a team of USVs has been investigated so far. Suppose that $I=(i_{1},i_{2},\ldots i_{m})$ represents *m* intruders trapped inside the bounded region R where m<n. To propose a decision making strategy for the USVs to choose the most dangerous intruder $i_{d}\in I$ at any time $t^{*}\in t$, we define the following distances:

(21) $$\begin{array}{c} d_{j}\left(t\right)=\left[x_{j}\left(t\right),P_{1}\right]\;\;\;for\;\;\;d_{i}\left(t\right)\in D\;\;\;for\;j=1,\ldots ,m\\ l_{c}i\left(t\right)=\left[c_{i}\left(t\right),P_{1}\right]\;\;\;for\;\;\;l_{c}i\left(t\right)\in Q\;\;\;for\;i=1,\ldots ,n. \end{array}$$

where $d_{i}\left(t\right)$ represents the distance between intruder *i* and point $P_{1}$ and $l_{c}\left(t\right)$ denotes the distance between USV *i* and point $P_{1}$. Furthermore, *D* and *Q* represent sets of distances between the intruders and the robots to the point $P_{1}$, respectively.

Now we introduce a decision making strategy set *F* for the USVs to choose the most dangerous intruder in any time $t^{*}\in t$ as follows:

(22) $$\begin{array}{c} f_{i}^{j^{\prime }}\left(t^{*}\right)=d_{j^{\prime }}\left(t^{*}\right)\;\;\;|\;\;\;\forall d_{j}\left(t^{*}\right)\in D\;\;\;\exists !\;\;d_{j^{\prime }}\left(t^{*}\right)<d_{j}\left(t^{*}\right)\;\;\;,j^{\prime }\in j=\left(1,\ldots ,m\right)\\ if\;\;i=1\\ f_{i}^{j^{\prime \prime }}\left(t^{*}\right)=d_{j^{\prime \prime }}\left(t^{*}\right)\;\;\;|\;\;\;\forall d_{j}\left(t^{*}\right)\in D\;\;\;\exists !\;\;d_{j^{\prime \prime }}\left(t^{*}\right)>d_{j}\left(t^{*}\right)\;\;\;,j^{\prime \prime }\in j=\left(1,\ldots ,m\right)\\ if\;\;i=n\\ f_{i}^{j^{\prime \prime \prime }}\left(t^{*}\right)=\inf\left|l_{c}i\left(t^{*}\right)-d_{j^{\prime \prime \prime }}\left(t^{*}\right)\right|\;\;\;,j^{\prime \prime \prime }\in j=\left(1,\ldots ,m\right)\\ if\;\;1<i<n. \end{array}$$

Then we introduce a set $ℵ=(H^{1},H^{2},\ldots ,H^{j})$ that includes a finite number of subsets $H^{j}$ in which j=1,2,…,m and each subset $H^{j}$ is supposed to be a set of numbers $H_{i}^{j-}\left(t^{*}\right),H_{i}^{j+}\left(t^{*}\right)$ in which i=k,k+1,…,n−k+1.

**Figure 3:**
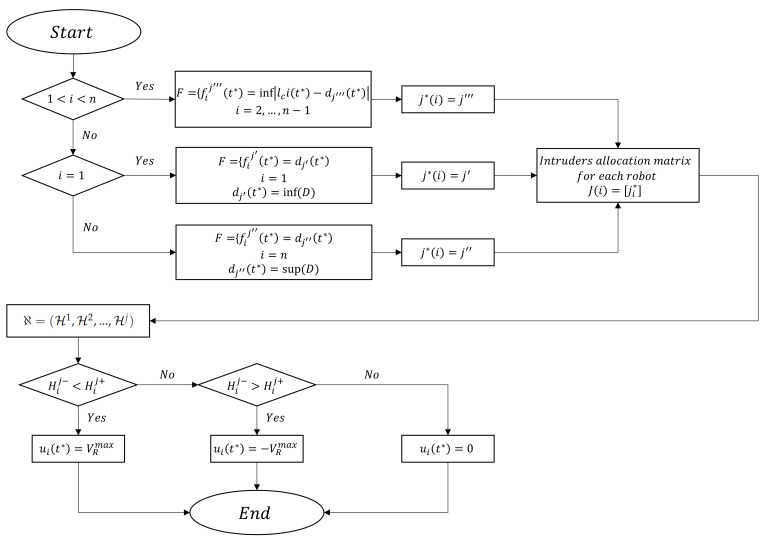
Decision making on multiple intruder detection.

Now by considering (22), each USV chooses the most dangerous intruder to intercept by taking a proper subset $H^{j}$ and using navigation law (17) for ϵ-intercepting the intruder *j*. Figure 3 illustrates the proposed methodology.

**Remark** **4.** *Notice that in this paper, we do not consider the problem of avoiding collisions between autonomous vehicles. However, this problem can be handled by combining the proposed method with various collision avoidance algorithms; see, for example* [41,42,43,44,45,46,47].

## 5. Simulation and Discussion

### 5.1. The Problem of Intercepting a Single Intruder

In this section, we present a simulation example that illustrates the main results of the paper using MATLAB R2020a. We considered a team of autonomous USVs that contains five individual point-wise USVs. The team members are deployed on the segment S, which represents the boundary of the region R. Each team member is responsible for intercepting the trapped intruder, which strives to escape the region through any interception point *P* on the boundary. We define the maximum velocity of the intruder as $V_{I}^{max}=4.2$ and the maximum velocity of each interceptor as $V_{R}^{max}=3.0$, which guarantees that Assumption 1 holds. Now, we apply Theorem 2 and the navigation law (17).

**Figure 4:**
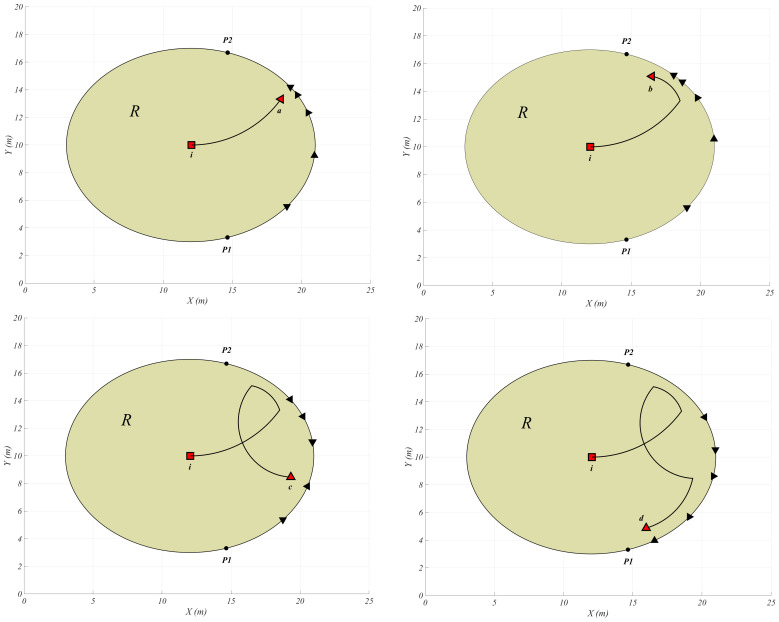
The intruder trajectory and the positions and the motion directions of the USVs when the intruder is at points a,b,c and *d*.

Figure 4 shows the positions and the motion directions of the USVs when the intruder tends to exit the region R and is at the points *a*, *b*, *c*, and *d*. The autonomous USVs are indexed in a counter-clockwise direction from point $P_{1}$ to point $P_{2}$. As it is obvious in Figure 4, the USVs 4 and 5 protect the dangerous point while the intruder *I* reaches point *a*. Then the team members are following the intruder while the intruder decided to change its direction towards point *b*. Then, after the USV 2 protects the dangerous point when the intruder reaches point *c* and finally the USV 1 intercepts the intruder at point *d*.

**Figure 5:**
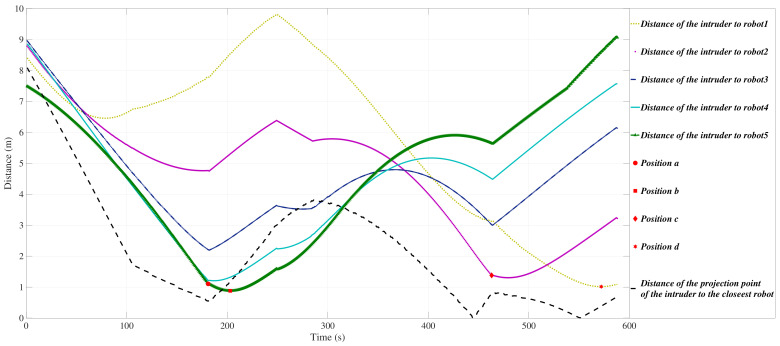
The evolution of the *y*-coordinates of the intruder and the USVs in segment *S*.

The evolution of the *y*-coordinates of the interceptors while the invasive intruder is moving along the trajectory is shown in Figure 5.

### 5.2. The Problem of Intercepting Multiple Intruders

To confirm the robustness and reliability of navigation law (17) as well as decision making strategy algorithm (22), a simulation scenario is presented for intercepting multiple intruders that are trapped in a region as they attempt to escape through some points $P_{k}^{j}$ in which $P_{1}<P_{k}^{j}<P_{2}$ and *j* represents the number of intruders. In this scenario, we consider five USVs moving along the border of the region R between points $P_{1}$ and $P_{2}$ and labelled 1, …, 5. We consider two intruders that attempt to escape the region through different points in the boundary. We assume that the robots are using onboard sensors (e.g., sonar) to avoid any collision and each team member maintains its position during the operation. We define the maximum velocity of the intruder as $V_{I}^{max}=4.2$ and the maximum velocity of each interceptor as $V_{R}^{max}=3.0$, which guarantees that Assumption 1 holds. Now, we apply Theorem 2, decision making strategy algorithm (22) and the navigation law (17).

**Figure 6:**
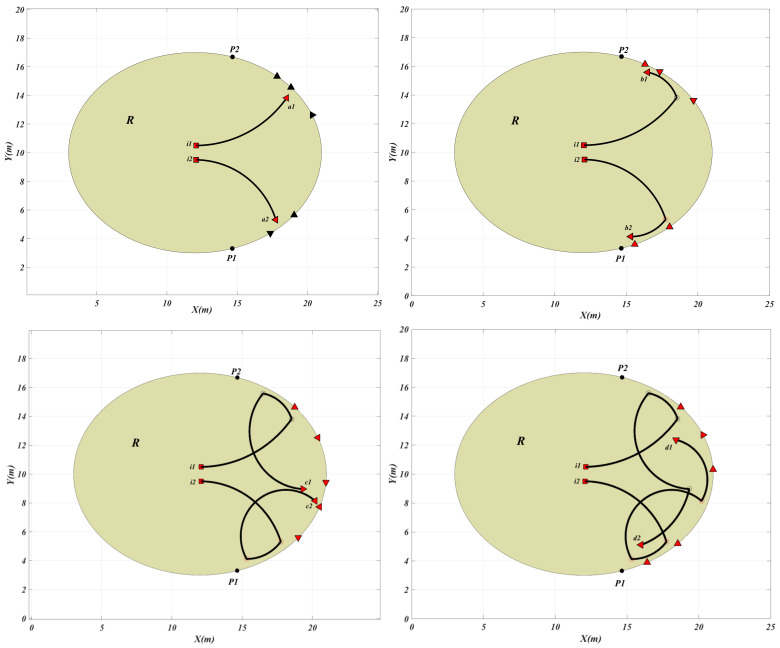
The intruder trajectory and the positions and the motion directions of the USVs when the intruder is at points a1,a2,b1,b2,c1,c2,d1 and d2.

Figure 6 shows the positions and the motion directions of the USVs when the intruder1 and intruder2 tend to exit the region R and are at the points (a1, a2), (b1, b2), (c1, c2), and (d1, d2), respectively. The autonomous USVs are indexed in a counter-clockwise direction from point $P_{1}$ to point $P_{2}$. As it is obvious in Figure 6, the USVs 4, 5 and 3 protect the dangerous point while the intruder1 i1 reaches point a1 as well as USVs 1, and 2 which are protecting the dangerous point while intruder2 i2 reaches the point a2. Then the team members follow the intruders while the intruders decide to change their direction towards point b1 and b2. Then after, the USVs 2 and 3 protect the dangerous points when the intruder1 and intruder2 reach point c1 and c2 and finally the USVs 4 and 5 intercept the intruder at point d1 while USV 1 intercepts the intruder2 at point d2.

**Figure 7:**
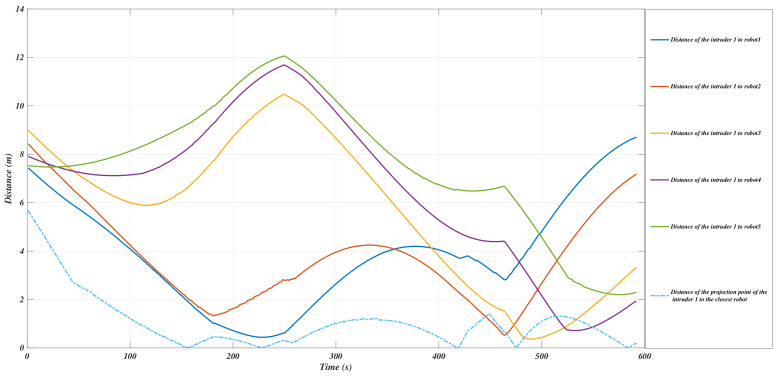
The evolution of the *y*-coordinates of the intruder1 and the USVs in segment *S*.

**Figure 8:**
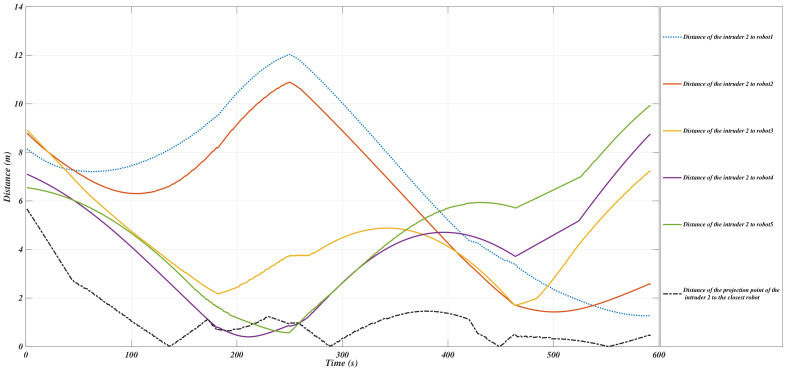
The evolution of the *y*-coordinates of the intruder2 and the USVs in segment *S*.

The evolution of the *Y*-coordinates of the interceptors while the invasive intruders are moving along the trajectories is shown in Figure 7 and Figure 8.

In conclusion, in this section, we have simulated the proposed decentralised autonomous navigation algorithm for scenarios with both a single intruder and multiple intruders. The results of computer simulations show the effectiveness of the developed navigation method.

## 6. Conclusions

A decentralised navigation control strategy for a group of autonomous USVs that are protecting a planar region by patrolling the boundary is presented in this study. On the other hand, there are trapped intruders that try to escape the region through some segments on the region’s boundary while trying to avoid being intercepted by the autonomous USVs. We derived a necessary and sufficient condition for the existence of a navigation algorithm that guarantees that the intruder is always intercepted on the boundary of the region for the case of a single intruder. The proposed navigation algorithm is based on some simple rules in which each autonomous USV only has information about the intruders and the closest team members. The proposed decentralised algorithm of autonomous navigation belongs to the class of sliding mode control laws. The effectiveness of the developed algorithm has been confirmed by computer simulations with both single and multiple intruders. An interesting possible future direction for this research will be to extend the obtained results to the case of ground unmanned autonomous vehicles moving not along a line in a perfect plane, but operating on uneven (very non-flat) terrains [48], or to the case of a team of aerial drones flying over uneven terrains [49]. In this case, a much more challenging problem of decentralised 3D autonomous navigation should be addressed. Another important direction of future research will be conducting real-world experiments with real USVs to evaluate the proposed navigation method.

## Data Availability

Not applicable.

## References

[1] Matveev A.S., Teimoori H., Savkin A.V. (2011). A method for guidance and control of an autonomous vehicle in problems of border patrolling and obstacle avoidance. Automatica.

[2] Matveev A.S., Wang C., Savkin A.V. (2012). Real-Time Navigation of Mobile Robots in Problems of Border Patrolling and Avoiding Collisions with Moving and Deforming Obstacles. Rob. Auton. Syst..

[3] Portugal D., Rocha R.P. (2013). Distributed Multi-Robot Patrol: A Scalable and Fault-Tolerant Framework. Rob. Auton. Syst..

[4] Marino A., Parker L.E., Antonelli G., Caccavale F. (2013). A decentralized architecture for multi-robot systems based on the null-space-behavioral control with application to multi-robot border patrolling. J. Intell. Robot Syst..

[5] Cheng T.M., Savkin A.V. (2009). A distributed self-deployment algorithm for the coverage of mobile wireless sensor networks. IEEE Commun. Lett..

[6] Savkin A.V., Cheng T.M., Xi Z., Javed F., Matveev A.S., Nguyen H. (2015). Decentralized Coverage Control Problems for Mobile Robotic Sensor and Actuator Networks.

[7] Cheng T.M., Savkin A.V., Javed F. (2011). Decentralized control of a group of mobile robots for deployment in sweep coverage. Rob. Auton. Syst..

[8] Cheng T.M., Savkin A.V. (2011). Decentralized control for mobile robotic sensor network self-deployment: Barrier and sweep coverage problems. Robotica.

[9] Semakova A.A., Ovchinnikov K.S., Matveev A.S. (2017). Self-deployment of mobile robotic networks: An algorithm for decentralized sweep boundary coverage. Robotica.

[10] Savkin A.V., Marzoughi A. Distributed control of a robotic network for protection of a region from intruders. Proceedings of the 2017 IEEE International Conference on Robotics and Biomimetics (ROBIO).

[11] Marzoughi A. Maximizing the probability of intrusion detection by a fleet of mobile robots using an intelligent game theoretic approach. Proceedings of the 2017 36th Chinese Control Conference (CCC).

[12] Polvara R., Sharma S., Wan J., Manning A., Sutton R. (2018). Obstacle avoidance approaches for autonomous navigation of unmanned surface vehicles. J. Navig..

[13] Xin J., Li S., Sheng J., Zhang Y., Cui Y. (2019). Application of improved particle swarm optimization for navigation of unmanned surface vehicles. Sensors.

[14] Mahacek P., Kitts C.A., Mas I. (2012). Dynamic guarding of marine assets through cluster control of automated surface vessel fleets. IEEE ASME Trans. Mechatron..

[15] Raboin E., Svec P., Nau D., Gupta S.K. Model-predictive target defense by team of unmanned surface vehicles operating in uncertain environments. Proceedings of the 2013 IEEE International Conference on Robotics and Automation.

[16] Raboin E., Svec P., Nau D.S., Gupta S.K. (2015). Model-predictive asset guarding by team of autonomous surface vehicles in environment with civilian boats. Auton. Robot..

[17] Simetti E., Turetta A., Casalino G., Storti E., Cresta M. (2010). Protecting assets within a civilian harbour through the use of a team of USVs: Interception of possible menaces. IARP Workshop on Robots for Risky Interventions and Environmental Surveillance-Maintenance (RISE-10).

[18] Li X., Huang H., Savkin A.V. (2020). A Novel Method for Protecting Swimmers and Surfers from Shark Attacks using Communicating Autonomous Drones. IEEE Internet Things J..

[19] Gage D.W. Command control for many-robot systems. Proceedings of the 19th Annual AUVS Technical Symposium.

[20] Sharma V., Patel R.B., Bhadauria H.S., Prasad D. (2016). Deployment schemes in wireless sensor network to achieve blanket coverage in large-scale open area: A review. Egypt. Inform. J..

[21] Huang H., Savkin A.V., Li X. (2020). Reactive Autonomous Navigation of UAVs for Dynamic Sensing Coverage of Mobile Ground Targets. Sensors.

[22] Isaacs R. (1999). Differential Games. A Mathematical Theory with Applications to Warfare and Pursuit, Control and Optimization.

[23] Yan R., Shi Z., Zhong Y. (2018). Reach-Avoid Games With Two Defenders and One Attacker: An Analytical Approach. IEEE Trans. Cybern..

[24] Chen M., Zhou Z., Tomlin C.J. Multiplayer reach-avoid games via low dimensional solutions and maximum matching. Proceedings of the American Control Conference.

[25] Shishika D., Kumar V. Local-game decomposition for multiplayer perimeter-defense problem. Proceedings of the IEEE Conference on Decision and Control.

[26] Savkin A.V., Huang H. (2020). Navigation of a Network of Aerial Drones for Monitoring a Frontier of a Moving Environmental Disaster Area. IEEE Syst. J..

[27] Cruz H., Eckert M., Meneses J., Martínez J.-F. (2016). Efficient forest fire detection index for application in unmanned aerial systems (UASs). Sensors.

[28] Odonkor P., Ball Z., Chowdhury S. (2019). Distributed operation of collaborating unmanned aerial vehicles for time-sensitive oil spill mapping. Swarm Evol. Comput..

[29] Jorge V.A., Granada R., Maidana R.G., Jurak D.A., Heck G., Negreiros A.P., dos Santos D.H., Gonçalves L.M., Amory A.M. (2019). A survey on unmanned surface vehicles for disaster robotics: Main challenges and directions. Sensors.

[30] Kum B.C., Shin D.H., Lee J.H., Moh T., Jang S., Lee S.Y., Cho J.H. (2018). Monitoring applications for multifunctional unmanned surface vehicles in marine coastal environments. J. Coast Res..

[31] Liu Z., Zhang Y., Yu X., Yuan C. (2016). Unmanned surface vehicles: An overview of developments and challenges. Annu. Rev. Control..

[32] Singh Y., Sharma S., Sutton R., Hatton D. (2018). Towards use of Dijkstra Algorithm for Optimal Navigation of an Unmanned Surface Vehicle in a Real-Time Marine Environment with results from Artificial Potential Field. TransNav.

[33] Campbell S., Naeem W., Irwin G.W. (2012). A review on improving the autonomy of unmanned surface vehicles through intelligent collision avoidance manoeuvres. Annu. Rev. Control.

[34] Savkin A.V., Huang H. (2019). Asymptotically optimal deployment of drones for surveillance and monitoring. Sensors.

[35] Cheng F., Yu W., Wan Y., Cao J. (2016). Distributed robust control for linear multiagent systems with intermittent communications and parameter uncertainties. IEEE Trans. Circuits Syst. II Express Briefs.

[36] Hong H., Yu W., Yu X., Wen G., Alsaedi A. (2017). Fixed-time connectivity-preserving distributed average tracking for multiagent systems. IEEE Trans. Circuits Syst. II Express Briefs.

[37] Skafidas E., Evans R.J., Savkin A.V., Petersen I.R. (1999). Stability results for switched controller systems. Automatica.

[38] Utkin V.I. (2013). Sliding Modes in Control and Optimization.

[39] Savkin A.V., Evans R.J. (2002). Hybrid Dynamical Systems. Controller and Sensor Switching Problems.

[40] Petersen I.R., Ugrinovskii V.A., Savkin A.V. (2000). Robust Control Design Using H-Infinity Methods.

[41] Savkin A.V., Wang C. (2013). A Simple Biologically-Inspired Algorithm for Collision Free Navigation of a Unicycle-like Robot in Dynamic Environments with Moving Obstacles. Robotica.

[42] Matveev A.S., Savkin A.V., Hoy M., Wang C. (2015). Safe Robot Navigation among Moving and Steady Obstacles.

[43] Hoy M., Matveev A.S., Savkin A.V. (2015). Algorithms for Collision Free Navigation of Mobile Robots in Complex Cluttered Environments: A Survey. Robotica.

[44] Marzoughi A. Decentralised Navigation Control of a Multi-Robot Team to Minimising Energy Consumption in an Unknown Obstacle-Ridden Area. Proceedings of the 2018 37th Chinese Control Conference (CCC).

[45] Marzoughi A. (2019). Switching Navigation for a Fleet of Mobile Robots in Multi-Obstacle Regions. Int. J. Mech. Eng. Robot. Res..

[46] Marzoughi A. Navigating a mobile robot to avoid moving obstacles using virtual source/sink force field. Proceedings of the 2017 IEEE International Conference on Robotics and Biomimetics (ROBIO).

[47] Marzoughi A. A decentralized position estimation switching algorithm to avoid a convex obstacle. Proceedings of the 2017 36th Chinese Control Conference (CCC).

[48] Ganganath N., Yuan W., Fernando T., Iu H.H.C., Cheng C.-T. (2018). Energy-efficient anti-flocking control for mobile sensor networks on uneven terrains. IEEE Trans. Circuits Syst. II Express Briefs.

[49] Savkin A.V., Huang H. (2019). Proactive deployment of aerial drones for coverage over very uneven terrains: A version of the 3D art gallery problem. Sensors.

